# Fast randomized approximate string matching with succinct hash data structures

**DOI:** 10.1186/1471-2105-16-S9-S4

**Published:** 2015-06-01

**Authors:** Alberto Policriti, Nicola Prezza

**Affiliations:** 1 Department of Mathematics and Informatics, University of Udine, via delle Scienze, 33100, Udine, Italy; 2 Institute of Applied Genomics, via J. Linussio, 33100 Udine, Italy

**Keywords:** Hashing, succinct indexing, BWT, de Bruijn property, complexity analysis

## Abstract

**Background:**

The high throughput of modern NGS sequencers coupled with the huge sizes of genomes currently analysed, poses always higher algorithmic challenges to align short reads quickly and accurately against a reference sequence. A crucial, additional, requirement is that the data structures used should be *light*. The available modern solutions usually are a compromise between the mentioned constraints: in particular, indexes based on the Burrows-Wheeler transform offer reduced memory requirements at the price of lower sensitivity, while hash-based text indexes guarantee high sensitivity at the price of significant memory consumption.

**Methods:**

In this work we describe a technique that permits to attain the advantages granted by both classes of indexes. This is achieved using Hamming-aware hash functions--hash functions designed to search the entire Hamming sphere in reduced time--which are also homomorphisms on de Bruijn graphs. We show that, using this particular class of hash functions, the corresponding hash index can be represented in linear space introducing only a logarithmic slowdown (in the query length) for the lookup operation. We point out that our data structure reaches its goals *without *compressing its input: another positive feature, as in biological applications data is often very close to be un-compressible.

**Results:**

The new data structure introduced in this work is called *dB-hash *and we show how its implementation--BW-ERNE--maintains the high sensitivity and speed of its (hash-based) predecessor ERNE, while drastically reducing space consumption. Extensive comparison experiments conducted with several popular alignment tools on both simulated and real NGS data, show, finally, that BW-ERNE is able to attain both the positive features of succinct data structures (that is, small space) and hash indexes (that is, sensitivity).

**Conclusions:**

In applications where space and speed are both a concern, standard methods often sacrifice accuracy to obtain competitive throughputs and memory footprints. In this work we show that, combining hashing and succinct indexing techniques, we can attain good performances and accuracy with a memory footprint comparable to that of the most popular compressed indexes.

## Background

The advent of New Generation Sequencing (NGS) technologies opened a new era in the field of DNA sequencing, providing researchers with powerful instruments able to produce millions of short (and, lately, long) reads per single run. This technology breakthrough poses considerable computational challenges since the sequenced fragments need to be quickly aligned--usually admitting errors--against genomes whose size is often of the order of giga-bases. From the algorithmic point of view, the problem of indexing texts to support pattern matching in the big-data domain is receiving significant attention, also due to the recent computational breakthroughs in the fields of succinct and compressed text indexes (a typical example is the FM self-index [1] used nowadays by many aligners, e.g. Bowtie [2]). Even though these important results solved most of the problems related to *exact *pattern matching, the problem of indexing a text with a succinct--or compressed--data structure that supports *inexact *pattern matching, still represents a considerable challenge. Due to the fact that BWT indexes are natively designed for exact pattern matching, in practice this problem is solved by splitting the pattern in fragments (e.g. using q-grams), searching for all possible variants of the modified pattern (e.g. by backtracking), or mixing the two strategies (hybrid). SOAP2 [3] adopts the first strategy, splitting the pattern in *k *+ 1 blocks, that is admitting at most *k *errors, and searching for exact occurrences of the blocks. The strategy is then completed by accelerating the search using a hash table to pre-compute backward search results on the BWT reference index. Bowtie [2] adopts a backtracking strategy on the FM index, inserting a mismatch in correspondence to low-quality bases during backward search. BWA [4] adopts a backtracking strategy on a BWT-based index that allows to (recursively) retrieve the occurrences of the pattern once an upper bound to the number of admitted mismatches is fixed. ERNE [5,6] implements a hybrid strategy, splitting the pattern in *t *blocks and computing, for each of them, its hash value. The particular class of hash functions employed (Hamming-aware hash function) allows to compute efficiently fingerprints of blocks at Hamming distance at most *k/t *from the original block. These fingerprints are then finally searched in the hash index. We mention also a similar hybrid strategy, based on *perfect Hamming codes*, adopted in [7].

One of the strengths of BWT-based tools is their reduced space requirement. The space for the data structure is often close to that required by the reference string (the genome). As an example, Bowtie requires only 2.7GB of RAM to index the Human genome. On the other hand, hash-based tools such as ERNE or SOAP [8] require much more memory to store their indexes, due to the fact that they need to explicitly memorize pointers to the reference. ERNE and SOAP require 19GB and 14GB of RAM to index the Human genome, respectively.

From a theoretical point of view, indexing for approximate string matching is still at an early research stage: even the most efficient solutions are, in practice, far from being usable. As a matter of fact, the most advanced results to date are able to guarantee efficiency on *either *space *or *speed. Not both at the same time. Letting *m *and *k *be the query length and the maximum number of allowed errors, respectively, simple backtracking strategies have a complexity that rapidly blows up with a factor of *m^k^*: impractical for searching with a reasonable amount of errors on (even not exceedingly) long patterns. Other solutions improve query time by trading on space requirements: letting *n *be the text length, the index of Cole et al. in [9] solves the problem in time  O((log *n*)*k *log log *n *+ *m *+ *occ*) and space  O(*n*(log *n*)*^k+1^*) bits, which in practice is too much even for small pattern lengths and number of errors. The solution of Chan et al. presented in [10] improves on space consumption requiring  O(*n *log *n*) bits, but query time increases to  O(*m *+ *occ *+ (*c *log *n*)*^k(*k*+1) ^*log log *n*), which exponentially blows up with the *square *of the number of errors.

We tackled the problem with a hash-based randomized algorithm that is able to reach expected fast performances and requires linear space. The two goals are obtained providing a hash function belonging to two particular classes of hash functions at the same time. The two classes are Hamming-aware and de Bruijn hash functions, respectively. Functions in the former class allow to "squeeze" the Hamming sphere of radius *k *centered at the query pattern *P*, to a Hamming sphere of radius  O(*k*) centered at the hash value of the query. Functions in the second class (de Bruijn) are homomorphisms on de Bruijn graphs. We show that their corresponding hash indexes can be represented in linear space introducing only a small slowdown of  O(log *m*) in the lookup operation.

We call *dB-hash *the resulting *succinct hash *data structure.

Letting *n *and *m *be the sizes of text and pattern, respectively, our algorithm reaches an expected time complexity of  O((log *n*)*^k^*log *m *+ *m*) while requiring  O(*n *log *σ*) bits (*σ *being the alphabet size) of space for the index only. Our result extends the strategy presented in [5,6], substituting a standard hash index with the new proposed dB-hash data structure and allowing us to improve on both time and space complexities with respect to [5,6]. Under the hypothesis that the indexed text is perturbed by random noise (e.g. genetic mutations in DNA), our algorithm improves upon theoretical linear-space upper-bounds discussed in the literature (a full formal proof of this can be found in [11]).

The resulting algorithm has been implemented in the short-reads aligner BW-ERNE, the natural adaptation of the hash-based ERNE aligner (ERNE-MAP) to the dB-hash data structure. Aim of this paper is to compare the performances of BW-ERNE with those of state-of-the-art short-read aligners. BW-ERNE uses a succinct dB-hash index based on the Burrows-Wheeler transform coupled with wavelet tree and not using any kind of compression (more on this aspect later). Moreover, the implementation takes into account biological information--such as base quality values--to improve performances without any significant loss in sensitivity.

Experimental results on the Vitis vinifera and Human genomes show that the dB-hash requires from 4 to 8 times less space than the standard hash used by ERNE (see Section). Tests run on both simulated and real reads show, in addition, that BW-ERNE maintains the same sensitivity of ERNE, improving also its throughput if reliable base qualities are available.

## Methods

We begin to illustrate our general strategy, showing how to represent a hash index in succinct space by building a *compact *( O(*n *log *σ*) bits) representation of all the fingerprints of length-*m *text substrings. A succinct index is then built over this representation, obtaining a succinct hash data structure.

### Definitions

Throughout this paper we will work with the alphabet Σ*_DN A _*= {*A, C, G, T, N*, $} (with *N *and $ being the undefined base and the contig end-marker, respectively) which, in practice, will be encoded in Σ*_DN A' _*= {0, 1, 2, 3} assigning a numerical value in Σ*_DN A' _*to *N *and $ characters. The size of our alphabet is, therefore, *σ *= |Σ*_DN A'_*| = 4, while with *n*, *m*, and *w *we will denote the reference length, the pattern length, and the (fixed) size of a computer memory-word (i.e. the number *σ^w ^*− 1 is assumed to fit in a memory word), respectively. As hash functions we will use functions of the form *h *: Σ*^m ^*→ Σ*^w ^*mapping length-*m *Σ-strings to length-*w *Σ-strings. If necessary, we will use the symbol ${\;}_{w}^{m}h$ instead of *h *when we need to be clear on *h*'s domain and codomain sizes. Given a string $P\in \overset{m}{\sum }$, the value $h\left(P\right)\in \overset{m}{\sum }$ will be also dubbed the *fingerprint *of P (in Σ*^w^*). With $T\in \sum^{n}$ we will denote the *text *that we want to index using our data structure. $T_{i}^{j}$ will denote T[i,...,i+j-1], i.e. the length-*j *prefix of the *i*-th suffix of *T*. A hash data structure *H *for the text *T *with hash function *h*, will be a set of ordered pairs (an index) such that $H=\left\{\left\langle h\left(T_{i}^{m}\right),i\right\rangle :0\leq i\leq n-m\right\}$, that can be used to store and retrieve the positions of length-*m *substrings of *T *(*m *is therefore fixed once the index is built). A *lookup *operation on the hash *H *given the fingerprint *h*(*P*), will consist in the retrieval of all the positions 0≤i<n such that $\left\langle h\left(P\right),i\right\rangle \in H$ and cases where $\left\langle h\left(P\right),i\right\rangle \in H$ but $T_{i}^{m}\neq P$ will be referred to as *false positives*.

The symbol ⊕ represents the exclusive OR (XOR) bitwise operator. *a *⊕ *b *where a,b∈∑, will indicate the bitwise XOR among the bits of the binary encoding of *a *and *b*. Analogously, *x *⊕ *y*, where $x,y\in \sum^{m}$ will indicate the bitwise XOR operation among the bits of the binary encoding of the two words *x *and *y *and V will denote the bitwise OR operator. $d_{H}\left(x,y\right)$ is the Hamming distance between $x,y\in \sum^{m}$. We point out that the Hamming distance is computed between characters in Σ, and not between the bits of the binary encoding of each of them. Patterns and fingerprints are viewed as bit vectors only when computing bitwise operations such as OR, AND, and XOR.

### Succinct representation of hash indexes

We begin by introducing the technique allowing succinct representation of hash indexes. The central property of the class of hash functions we are going to use is given by the following definition:

**Definition 1 ***Let *Σ = {0,..., |Σ| −1}. *We say that a function h *: Σ*^m ^*→ Σ*^w ^is a *de Bruijn *hash function if and only if, for every pair of strings P, Q *∈ Σ*^m^*

$$P_{1}^{m-1}=Q_{0}^{m-1}\Rightarrow h{\left(P\right)}_{1}^{w-1}=h{\left(Q\right)}_{0}^{w-1}$$

With the following theorem we introduce the hash function used in the rest of our work and in the implementation of our structure:

**Theorem 1 ***Let P *∈ Σ*^m^*. *The hash function h*_⊕ _: Σ*^m ^*→ Σ*^w ^w *≤ *m defined as*

$$h⊕\left(P\right)=\left(\overset{\left\lceil m/w\right\rceil -2}{\underset{i=0}{⊕}}P_{i\;w}^{w}\right)⊕P_{m-w}^{w}$$

*is a de Bruijn hash function*.

A detailed proof of this theorem is given in Additional file 1. Given a de Bruijn hash function ${}_{w}^{m}h:\sum^{m}\to \sum^{w}$ we can "extend" it to another de Bruijn hash function ${}_{n-m+w}^{n}h:\overset{n}{\sum }\to \overset{n-m+w}{\sum }$, operating on input strings of length *n *greater than or equal to *m*, as follows:

**Definition 2 ***Given ${}_{w}^{m}h:\sum^{m}\to \sum^{w}$ de Bruijn hash function and n *≥ *m, the hash value of ${}_{n-m+w}^{n}h$ on T *∈ Σ*^n^, is the unique string ${}_{n-m+w}^{n}h\left(T\right)\in \overset{n-m+w}{\sum }$ such that:*

$${}_{n-m+w}^{n}h{\left(T\right)}_{i}^{w}{=}_{w}^{m}h\left(T_{i}^{m}\right),$$

*for every *0 ≤ *i *≤ *n *− *m*.

It is easy to show that a function enjoying the property in Definition 2 is a homomorphism on de Bruijn graphs (having as sets of nodes Σ*^m ^*and Σ*^w^*, respectively). Since ${}_{w}^{m}h$ univocally determines ${}_{n-m+w}^{n}h$ and the two functions coincide on the common part Σ*^m ^*of their domain, in what follows we will simply use the symbol *h *to indicate both.

From Definitions 1 and 2 we can immediately derive the following important property:

**Lemma 1 ***If h is a de Bruijn hash function, n *≥ *m, and P *∈ Σ*^m ^occurs in T *∈ Σ*^n ^at position i, then h*(*P*) *occurs in h*(*T*) *at position i. The opposite implication does not (always) hold; we will refer to cases of the latter kind as false positives*.

On the ground of Lemma 1 we can propose, differently from standard approaches in the literature, to build an index *over the hash value of the text*, instead of building it over the text. This can be done while preserving our ability to locate substrings in the text, since we can simply turn our task into that of locating *fingerprints *in the hash of the text *T*. We call dB-hash the data structure obtained with this technique. Notice that the underlying hash data structure is simulated by searching the occurrences of *h*(*P*) in *h*(*T*) during a lookup operation, so the algorithm is transparent to the particular indexing technique used.

### Search algorithm

The core of our searching procedure is based on the algorithm *rNA *(Vezzi et al. [5], Policriti et al. [6]), a hash-based randomized numerical aligner based on the concept of *Hamming-aware *hash functions (see [5] and [6] for more details). Hamming-aware hash functions are particular hash functions capable to "squeeze" the Hamming ball of radius *k *around a pattern P to a Hamming ball of the same radius around the hash value of *P*. This feature allows to search the entire Hamming ball around *P *much more efficiently. The following theorem holds:

**Theorem 2 ***The de Bruijn function h*_⊕ _*defined in Definition 1 is a Hamming aware hash function. In particular:*

$$d_{H}\left(P,P\prime \right)\leq k\Rightarrow d_{H}\left(h_{⊕}\left(P\right),h_{⊕}\left(P\prime \right)\right)\leq 2k$$

for every $P,P\prime \in \sum_{DN\;A\prime }^{m}$

See Additional file 1 for a detailed proof of this theorem. Since *h*_⊕ _is a de Bruijn *and *Hamming aware hash function, we can use it to build our structure and adapt the rNA algorithm to it. We call dB-rNA the new version of the rNA algorithm adapted to the dB hash data structure. More in detail, the Hamming-awareness property of *h*_⊕ _guarantees that, given a pattern *P *to be searched in the index, the set {*h*_⊕_(*P'*) : *d_H_*(*P, P'*) ≤ *k*} is small-- O((2*σ *− 2)*^k ^w^k^*) =  O(6*^k ^w^k^*) elements in our application--and can be computed in time proportional to its size. Notice that, with a generic hash function *h*, only the trivial upper bound  O((*σ *− 1)*^k ^m^k^*) can be given to the size of this set since each different *P'* such that *d_H_*(*P, P'*) ≤ *k *could give rise to a distinct fingerprint. Our proposed algorithm is almost the same as the one described in [5,6], the only difference being that the underlying data structure is a dB-hash instead of a standard hash. Briefly, the search proceeds in 3 steps. For each pattern *P*:

1 *h*_⊕_(*P*) is computed;

2 the index is searched for each element in the set $\left\{h\left(P\prime \right):d_{H}\left(P,P\prime \right)\leq k\right\}$,

3 for each occurrence found, the text and the pattern *P *are compared to determine Hamming distance and discard false positives.

In practice, in our implementation we also split the pattern *P *in non-overlapping blocks before searching the index. With this strategy we reduce the maximum number of errors to be searched, improving the speed of the tool.

### Complexity analysis

Let *occ *be the number of occurrences with at most *k *errors of the searched pattern *P *in *T*. Assuming that the alphabet size *σ *is a power of 2 (condition satisfied in our application), the expected complexity on uniformly distributed inputs of our algorithm has an upper bound of

$$O\left({\left(2\sigma \right)}^{k}{\left(\text{log}n\right)}^{k}\text{log}m+\left(occ+1\right)\cdot m\right),$$

here, *σ *= 4 is the size of the alphabet Σ*_DN A'_*. A fully formal proof of (an extended version of) this analysis can be found in [11].

### Quality-aware strategy

Our tool implements a quality-aware heuristic that significantly improves search speed, at the price of a small loss in sensitivity. Briefly, we use base qualities to pick up only a small fraction of the elements from the Hamming ball centred on the hash *h*(*B*) of the searched block *B*, following the assumption that a high quality base is unlikely to be a miscall. Since in practice we divide the read in non-overlapping blocks and the heuristic affects only the searched block, with this strategy we lose only a small fraction of single variants like SNPs. More in detail, let $Q\in {\mathbb{N}}^{m}$ be (e.g.) the Phred quality (see [12]) string associated to the searched block *B*. We compute a hash value on *Q *using the following hash function:

**Definition 3 **With $h_{\vee }:{\mathbb{N}}^{m}\to {\left\{0,3\right\}}^{w}$ we indicate the hash function defined as

$$h_{\vee }\left(Q\right)=\left(\vee_{i=0}^{\left\lceil m/w\right\rceil -2}f_{q}\left(Q_{iw}^{w}\right)\right)\vee f_{q}\left(Q_{m-w}^{w}\right)$$

where $f_{q}:{\mathbb{N}}^{w}\to {\left\{0,3\right\}}^{w}$ is defined as

$$f_{q}\left(Q\right)\left[i\right]=\left\{\begin{array}{c} 0\;if\;Q\left[i\right]>q\\ 3\;\text{otherwise} \end{array}\;,\;i=0,...,w-1\right)$$

*q *is a quality threshold (in our implementation we use *q *= 15). The values 0 and 3 have been chosen due to their binary representation (00 and 11, respectively). If $h_{\vee }\left(Q\right)\left[i\right]=3$, then during search we try to insert an error in position *i *of *h*_⊕ _(*B*) since at least one of the bases used to compute *h*_⊕ _(*B*)[*i*] has a low-quality. The quality-aware strategy is then implemented as follows: let *B *be the block to be searched and *Q *its associated Phred quality string. A fingerprint *f *(representing a block at distance at most *k *from *B*) is searched in the structure if and only if $\left(f⊕h_{⊕}\left(B\right)\right)\vee h_{\vee }\left(Q\right)=h_{\vee }\left(Q\right)$, i.e. if *f *differs from *h*_⊕_(*B*) only in positions corresponding to low quality bases. Since the number of low-quality base pairs in a read is typically low, this strategy allows to drastically reduce search space (which in practice leads approximately to a 10x speedup) if reliable qualities are available. In the results section we show, moreover, that this strategy has only a negligible impact on SNP detection and on the overall precision of our tool.

### BW-ERNE: implementation details

We implemented our algorithm and data structure in the short reads aligner BW-ERNE (*Burrows-Wheeler Extended Randomized Numerical alignEr*), downloadable at http://erne.sourceforge.net. As hash function for our index we use *h*_⊕_. Given a text $T\in \sum_{DN\;A}^{n}$, we calculate *h*_⊕ _(*T*)*^BW T^*--the Burrows-Wheeler transform of *h*_⊕_(*T*)--adding the necessary additional structures needed to perform backward search and to retrieve text positions from *h*_⊕ _(T)*^BW T ^*positions. BW-ERNE includes (from its predecessor ERNE) also a simple and fast strategy to allow a single indel in the alignment. This strategy does not affect running times and permits to correctly align a large fraction of short reads that come with indels (see Results section). It is well known that DNA is extremely difficult to compress and for this reason we choose not to introduce compression in our structure. Even if our index is not compressed, experiments show (see Section) that its memory requirements are similar or even smaller than those of other tools based on the FM index such as Bowtie [2], BWA [4] and SOAP2 [3]. Briefly, the structure is composed by three parts: the index, the plain text, and an auxiliary (standard) hash.

#### BWT index

The BWT index is constituted by $h_{⊕}{\left(T\right)}^{BWT}$ stored as a wavelet tree (*n *log *σ *bits), *rank *counters (*o*(*n *log *σ*) bits), and sampled suffix array pointers for its navigation (*n *+ *o*(*n*)) bits for one rank structure and 2*n *bits for one SA pointer every 16 text positions; the user can however modify the SA pointers density).

#### Plain text

$T\in \sum_{DN\;A}^{n}$ is stored in a 3-bits per base format in blocks of 8 symbols (3*n *bits). We exploit this encoding to perform  O(1) text-query comparison of a single block, improving the speed of the algorithm.

#### Auxiliary hash

To speed up lookup operations, we finally store an auxiliary hash *H^AU X ^*that indexes the *w_aux _*most significant digits of the fingerprints: the intervals obtained by backward search on all the numbers in the set $\left\{0,...,\sigma^{w_{aux}}-1\right\}$ are precomputed and stored in *H^AU X^*. In this way, a lookup operation on *H^BW T ^*requires one lookup in *H^AU X ^*followed by *w *− *w_aux _*steps of backward search. We require *H^AU X ^*to occupy only *n *bits. This limit gives us an upper bound for *w_aux _*of ${\text{log}}_{\sigma }n-{\text{log}}_{\sigma }{\text{log}}_{n}$. It can be proved [11] that the optimal word size for our algorithm is $w={\text{log}}_{\sigma }\left(mn\right)$. Combining these results it follows that the cost of a lookup operation in our data structure is  O(log *m*).

Summing up, the total space occupancy of the dB-hash data structure implemented in BW-ERNE is of 2nlogσ+4n+o(nlogσ) bits, corresponding in practice to approximately 1.4*n *Bytes (this fraction may slightly vary for different reference sizes): the index is succinct.

## Results

In order to assess the performances of BW-ERNE, we performed extensive experiments on two genomes: *Vitis vinifera *(480 Mbp) and *Human genome *(hg19 reference, 3.2 Gbp).

Simulations on Vitis vinifera were used to compare the alignment accuracy and speed of BW-ERNE, its (old) standard-hash counterpart ERNE, Bowtie, BWA, and SOAP2, in presence of reliable base qualities on a medium-sized genome. Hence, in order to precisely asses the correctness of our results, we used simulated data produced by the SimSeq simulator, https://github.com/jstjohn/SimSeq. Experiments on real data (not reported here as subsumed by the experiments on human) confirm the conclusions.

Experiments on the Human genome, instead, were performed in order to assess the precision of BW-ERNE in absence of base quality information (we per-formed a GCAT test, http://www.bioplanet.com/gcat/), to assess the impact of BW-ERNE's quality-aware strategy on SNPs detection (using SimSeq and random SNP simulation), and to assess the performances of our aligner on a real 10x coverage Illumina library downloaded from the 1000genomes project's database (http://www.1000genomes.org/).

All experiments were performed on a intel core i7 machine with 12 GB of RAM running Ubuntu 14.04 operating system. See Additional file 2 and Additional file 3 for further informations about the implementation usage and the commands used to perform the experiments, respectively.

### Memory footprint of the indexes

**Figure 1:**
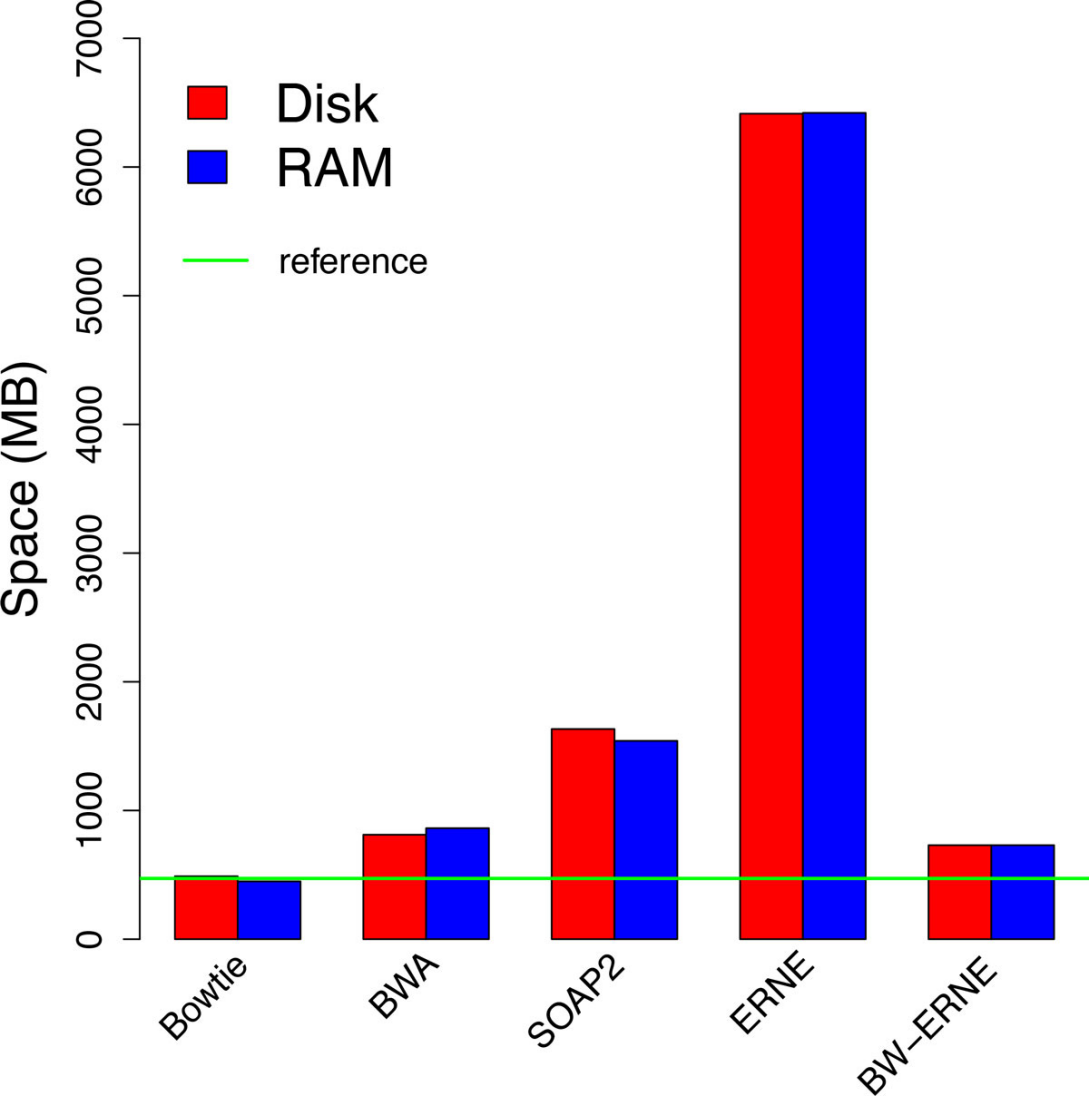
**Space required by the tested tools (Vitis Vinifera genome) This plot compares the space needed by some of the most popular short-read aligners to index the Vitis Vinifera genome**. We reported space on disk (storage of the index) and RAM (structures loaded in memory). Full text indexes such as the hash data structure implemented in ERNE require much more space than the succinct and compressed indexed used by the other tools. Notice that the space required by succinct (BW-ERNE) and compressed (Bowtie, SOAP2, BWA) indexes is almost the same in DNA indexing: this is due to the fact that DNA is, in general, extremely difficult to compress.

BW-ERNE significantly reduces the space requirements of ERNE, requiring approximately 8.7 times less space on the Vitis Vinifera genome (730 MB vs 64 GB) and approximately 4.4 times less space on the Human genome (4.3 GB vs 19 GB). The plot in Figure 1 compares the different indexes sizes on Vitis Vinifera and gives a clear idea of the most important difference between full-text and succinct indexes, the former requiring space proportional to *n *log *n *(*n *pointers to the text) while the latter is using an amount of space close to that necessary for the plain text. More-over, differences among BWT-based tools are minimal (few hundred MB) if compared to the gap between hash-based (ERNE) and BWT-based (Bowtie, BWA, SOAP2, BW-ERNE) aligners. Among the tested tools only Bowtie requires less space than our tool, even if in the dB-hash data structure we do not perform any data compression. This is due to the fact that DNA is a high-entropy string and, therefore, almost incompressible using standard techniques such as the ones implemented in the FM index. Finally, the horizontal green line, marking the size of the reference fasta file, gives an idea of how efficiently can succinct and compressed indexes represent their structures: in particular, Bowtie is able to reach a RAM memory footprint that is *smaller *than that of the reference file itself, while BWA and BW-ERNE require slightly more space. On the Human genome, BW-ERNE index requires only 4.3 GB of space to be stored and loaded during alignment. This is slightly more than the indexes of Bowtie and BWA (2.7 GB and 3.2 GB of RAM, respectively), but considerably less than ERNE's hash table, which requires 19 GB of space to be stored and loaded during alignment.

### Simulated data with reliable base qualities

In this experiment we compared the tools on 5M of 100bp single-end reads with simulated base qualities. This dataset was generated from the Vitis Vinifera genome using the SimSeq simulator, adopting the built-in Illumina error model. The number of correctly mapped reads was estimated comparing the bam files generated by SimSeq and the aligners (reads mapping in multiple locations were evaluated on the unique reported alignment).

**Figure 2:**
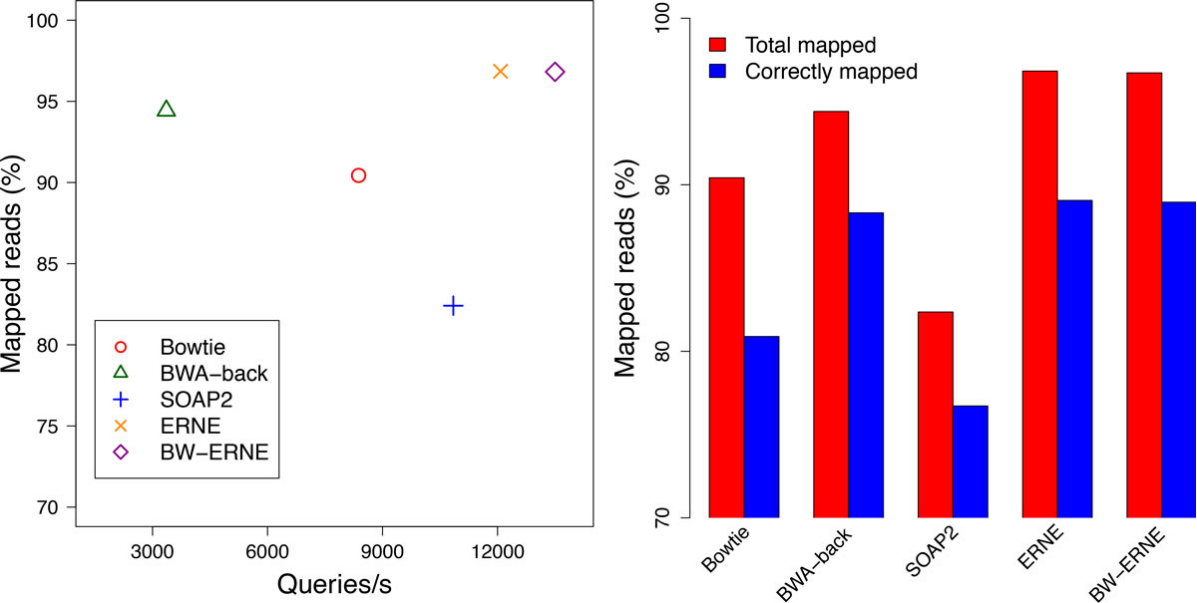
**Results on 5M 100 bp single-end reads simulated using the tool SimSeq (Vitis Vinifera genome) These experiments allowed us to judge how the presence of reliable base qualities affected the quality-aware strategies of Bowtie and BW-ERNE**. The left plot shows that BW-ERNE is able to exploit at best the presence of reliable base qualities: our tool was several times faster than the other tools, while at the same time correctly aligning the highest number of reads (together with ERNE).

The plots in Figure 2 show that BW-ERNE is able to exploit at best quality information without losing accuracy with respect to ERNE while, at the same time, improving significantly its performances. BWERNE was executed with default settings and using 1 thread. The plot on the left hand side of Figure 2 shows that BW-ERNE was 2 times faster than Bowtie, and 4 times faster than BWA. This speed came with no penalties on the number of mapped reads, which was the highest among all tools, with BW-ERNE and ERNE aligning 97% of all reads, BWA 94%, Bowtie 90% and SOAP2 82%. Finally, the plot on the right hand side of Figure 2 shows the accuracy of the tools in terms of correctly mapped reads. The gap between mapped and correctly mapped reads is due to reads mapping in multiple locations, which were judged on the base of the unique reported alignment. The plot shows that ERNE and BW-ERNE were the most accurate tools, correctly aligning the highest number of reads.

### Simulated data without reliable base qualitie

A public GCAT experiment (Human genome) was performed in order to assess the precision of our tool in absence of reliable base qualities. The experiment consisted of 12M 100bp single-end reads with 0.02% small indel frequency. The results are available at the address http://www.bioplanet.com/gcat/reports/3705-muwwfqmbjb/alignment/100bp-se-small-indel/BW-ERNE/compare-26-27-38. In this experiment, BWERNE was executed with the option --sensitive, which ignores base qualities (not meaningful in the GCAT simulation), and using 4 threads. BW-ERNE completed the alignment in 1 hour and 28 minutes, for an overall throughput of 8M reads per hour (approximately 4x faster than with only 1 thread). The results are reported in Table 1: BW-ERNE was one of the most accurate aligners, correctly aligning 97.3% of all the reads. This fraction is comparable to that of Novoalign and BWA-mem, and higher than that of Bowtie2 and BWA.

**Table 1:** Results of the GCAT experiment (data coming from GCAT website).

Tool	Total Reads	Correct	Incorrect	Unmapped
BW-ERNE	11,945,249	97.30%	2.311%	0.3900%
Bowtie2	11,945,249	93.52%	5.284%	1.192%
Novoalign	11,945,249	97.47%	0.08329%	2.445%
Novoalign3	11,945,249	97.47%	0.08300%	2.442%
BWA	11,945,249	93.91%	1.707%	4.385%
BWA-SW	11,971,702	94.29%	4.139%	1.576%
BWA-MEM	11,951,583	97.47%	2.515%	0.01361%

BW-ERNE ranks among the most precise tools, correctly aligning a number of reads comparable to that of slower aligners such as Novoalign.

### Validation of the quality-aware strategy in presence of SNPs

In order to assess the impact of our quality-aware strategy on SNP detection, we simulated (using SimSeq) 10M of 100bp single-end reads, using as reference the Human genome (hg19). After the simulation, each base was randomly substituted with probability 0.005 to simulate SNPs (which, from the aligner's point of view, are simply mismatches with high base-quality). This strategy allowed us to track reads containing SNPs, permitting a separate verification of the alignment's correctness for reads with and without this kind of mutations. BW-ERNE was executed twice on the mutated dataset: with default settings (quality-aware strategy enabled) and with the --sensitive option enabled (quality-aware strategy disabled). An alignment was considered correct if and only if both chromosome and strand coincided with those outputted by SimSeq and if the alignment's position was within 50 bases from the position outputted by Simseq (in order to account for indels and clipped bases). Reads with multiple alignments were judged on the basis of their unique reported alignment. Of the 10M simulated reads, 39.42% contained at least one SNP. BWERNE in sensitive mode (quality-aware strategy disabled) correctly aligned 87.80% of the reads *without *SNPs and 87.64% of the reads *with *SNPs, thus showing (as expected) no significant bias towards reads without SNPs (the 0.16% difference can be explained with the fact that reads with SNPs are inherently more difficult to align). BW-ERNE with the quality-aware strategy enabled correctly aligned 86.54% of the reads *without *SNPs and 85.25% of the reads *with *SNPs, thus showing only a slight bias towards reads without SNPs.

### Real data -- Human genome

To conclude, the experiment on a real high-coverage Human Illumina library, allowed us to validate the results obtained on simulated reads and to assess the performances of BW-ERNE on large datasets. In this experiment we aligned 320M of 100bp pairedend reads, corresponding to a 10x coverage of the Human genome, downloaded from the 1000genomes project's database (top 160M reads in ftp://ftp. http://1000genomes.ebi.ac.uk/vol1/ftp/data/NA12878/sequence_read/SRR622457_1.filt.fastq.gz and top 160M reads in ftp://ftp.1000genomes.ebi.ac.uk/vol1/ftp/data/NA12878/sequence_read/SRR622457_2.filt.fastq.gz). BW-ERNE was executed with default settings (quality-aware strategy enabled) and using 4 threads. Our tool completed the alignment in 3 hours and 15 minutes, for an overall throughput of 98 millions of reads per hour. 15916364 reads (5% of all the reads) were automatically discarded by the builtin trimmer due to low base quality. Of the remaining 304083636 reads, 300717664 (98.9%) were successfully aligned to the reference and 3365972 (1.1%) were not found. Among the aligned reads, 284650317 (94.6%) were aligned in only one position and 16067347 (5.4%) in multiple positions.

## Conclusions

In this paper we presented a new technique that permits a succinct representation of hash indexes using hash functions with the property of being Hamming-aware and homomorphisms on de Bruijn graphs. We used this technique to build a succinct index--dubbed dB-hash--which, combined with a previously published hash-based algorithm, allowed us to lower the upper bound to the average-case complexity of the *k*-mismatch problem in succinct space. We implemented our algorithm and data structure in the short-reads aligner BW-ERNE. Tests on both simulated and real data, using the most popular short reads aligners, allowed us to validate also in practice the efficiency of our algorithm, which proved to be extremely accurate and fast, especially if reliable base qualities are available.

We are exploring numerous extensions of the work discussed here, on both the theoretical and practical side. From the theoretical point of view, we are studying ways to extend our complexity results to a more general analysis of hashing, which could turn out useful in the complexity analysis of hash-based algorithms. Other theoretical extensions of our work include the study of the properties of *h*_⊕ _as a *text transform*, randomizing the text and to be used in combination with existing pattern-matching algorithms. From the practical point of view, we are extending our BW-ERNE aligner with several new features such as long-reads alignment (combining the techniques discussed here with gapped strategies) and bisulfite-treated reads alignment (see [13]).

## Availability

ERNE (*Extended Randomized Numerical alignEr*, version 2) is a short string alignment package whose goal is to provide an all-inclusive set of tools to handle short reads. ERNE comprises: ERNE-MAP, ERNE-DMAP, ERNE-FILTER, ERNE-VISUAL, ERNE-BS5, and ERNE-METH. ERNE is free software and distributed with an Open Source License (GPL V3) and can be downloaded at: http://erne.sourceforge.net

## Competing interests

The authors declare that they have no competing interests.

## Authors' contributions

Both authors equally contributed to the idea and to the design of the algorithm and the experiments. NP developed the tool and performed the experiments. Both authors wrote the paper.

## Supplementary Material

Additional file 1 proofs of theorems file: additional file 1.pdf Click here for file

Additional file 2 implementation usage file: additional file 2.pdf Click here for file

Additional file 3 commands used to perform the experiments file: additional file 3.pdf Click here for file

## Declarations

The publication costs for this article were supported by the Italian EPIGEN Flagship Project.

This article has been published as part of *BMC Bioinformatics *Volume 16 Supplement 9, 2015: Proceedings of the Italian Society of Bioinformatics (BITS): Annual Meeting 2014: Bioinformatics. The full contents of the supplement are available online at http://www.biomedcentral.com/bmcbioinformatics/supplements/16/S9.

## References

[B1] Ferragina P Manzini G Opportunistic data structures with applications Foundations of Computer Science, 2000 Proceedings 41st Annual Symposium on 2000 390 398 IEEE

[B2] Langmead B Trapnell C Pop M Salzberg SL Ultrafast and memory-efficient alignment of short DNA sequences to the human genome Genome Biol 2009 10 3 R25 10.1186/gb-2009-10-3-r25 19261174 PMC2690996

[B3] Li R Yu C Li Y Lam TW Yiu SM Kristiansen K Wang J SOAP2: an improved ultrafast tool for short read alignment Bioinformatics 2009 25 15 1966 1967 10.1093/bioinformatics/btp336 19497933

[B4] Li H Durbin R Fast and accurate short read alignment with Burrows-Wheeler transform Bioinformatics 2009 25 14 1754 1760 10.1093/bioinformatics/btp324 19451168 PMC2705234

[B5] Vezzi F Del Fabbro C Tomescu AI Policriti A rNA: a fast and accurate short reads numerical aligner Bioinformatics 2012 28 123 124 10.1093/bioinformatics/btr617 22084252

[B6] Policriti A Tomescu AI Vezzi F A randomized Numerical Aligner (rNA) J Comput Syst Sci 2012 78 6 1868 1882 10.1016/j.jcss.2011.12.007

[B7] Takenaka Y Seno S Matsuda H Perfect Hamming code with a hash table for faster genome mapping BMC genomics 2011 12 Suppl 3 S8 10.1186/1471-2164-12-S3-S8 22369457 PMC3333191

[B8] Li R Li Y Kristiansen K Wang J SOAP: short oligonucleotide alignment program Bioinformatics 2008 24 5 713 714 10.1093/bioinformatics/btn025 18227114

[B9] Cole R Gottlieb LA Lewenstein M Dictionary matching and indexing with errors and don't cares Proceedings of the thirty-sixth annual ACM symposium on Theory of computing 2004 91 100 ACM

[B10] Chan HL Lam TW Sung WK Tam SL Wong SS A linear size index for approximate pattern matching Combinatorial Pattern Matching 2006 Springer 49 59

[B11] Policriti A Prezza N Hashing and Indexing: Succinct Data Structures and Smoothed Analysis Algorithms and Computation 2014 Springer 157 168

[B12] Ewing B Hillier L Wendl MC Green P Base-calling of automated sequencer traces usingPhred. I. Accuracy assessment Genome research 1998 8 3 175 185 10.1101/gr.8.3.175 9521921

[B13] Prezza N Del Fabbro C Vezzi F De Paoli E Policriti A ERNE-BS5: aligning BS-treated sequences by multiple hits on a 5-letters alphabet Proceedings of the ACM Conference on Bioinformatics, Computational Biology and Biomedicine 2012 12 19 ACM

