# Supplementary material for: Fast randomized approximate string matching with succinct hash data structures
Source: BMC Bioinformatics. 2015 Jun 1;16(Suppl 9):S4. doi: 10.1186/1471-2105-16-S9-S4 (PMC4464037; doi:10.1186/1471-2105-16-S9-S4)
Supplement: Additional file 1 — proofs of theorems file: additional file 1.pdf [file 1471-2105-16-S9-S4-S1.pdf]

Additional file 1 : proofs of theorems

Alberto Policriti[1,2] and Nicola Prezza[1]

[1] University of Udine, Department of Mathematics and Informatics, Udine, Italy
[2] Institute of applied genomics, Udine, Italy

1 $h_\oplus$ is a de Bruijn function

Theorem 1. *Let $\Sigma = \{0, ..., 2^r - 1\}, r > 0$, $P \in \Sigma^m$. The hash function $h_\oplus : \Sigma^m \rightarrow \Sigma^w$, $w \leq m$ defined as*

$$h_\oplus(P) = \left(\bigoplus_{i=0}^{\lceil m/w \rceil - 2} P^w_{iw} \right) \oplus P^w_{m-w}$$

is a de Bruijn hash function.

Proof. Taking the alphabet to be $\Sigma = \{0, ..., 2^r - 1\}, r > 0$ guarantees that $a \oplus b \in \Sigma \; \forall a, b \in \Sigma$, so that the codomain of the function is Σ^w. Let $\sigma_1, \sigma_2 \in \Sigma^m$ be such that $\sigma_1[1, ..., m-1] = \sigma_2[0, ..., m-2]$. The following lemma trivially holds:

Lemma 2. *If $\sigma_1[1, ..., m-1] = \sigma_2[0, ..., m-2]$, $\sigma_1, \sigma_2 \in \Sigma^m$ then*

$$\sigma_1[l, ..., r] = \sigma_2[l-1, ..., r-1] \quad \forall 0 < l \leq r < m$$

The bitwise $\oplus$ operator has the property that the i-th bit of $x_1 \oplus x_2,\ x_1, x_2 \in \Sigma^w$ depends only on the bits $x_1[i]$ and $x_2[i]$. This fact implies the following:

Lemma 3. *if $x_i \in \Sigma^w,\ i = 0, ..., k$ and $y = \bigoplus_{i=0}^k x_i$ then*

$$y[l, ..., r] = \bigoplus_{i=0}^{k} x_i[l, ..., r] \quad \forall 0 \leq l \leq r < w$$

From the definition of $h_\oplus$ and Lemma 3 we have that $h_\oplus(\sigma_1)[1, ..., w-1]$ is equal to

$$\left(\bigoplus_{i=0}^{\lceil m/w \rceil - 2} \sigma_1[iw+1, ..., iw+w-1] \right) \oplus \sigma_1[m-w+1, ..., m-1] \qquad (1)$$

Applying Lemma 2 we obtain that (1) is equal to

$$\left(\bigoplus_{i=0}^{\lceil m/w \rceil - 2} \sigma_2[iw, ..., iw+w-2] \right) \oplus \sigma_2[m-w, ..., m-2] \qquad (2)$$

Applying again the definition of $h_\oplus$ and Lemma 3 we finally obtain that (2) is equal to $h_\oplus(\sigma_2)[0, ..., w-2]$.

□

2 Hamming-awareness of $h_\oplus$

To prove the Hamming-awareness of $h_\oplus$, we firstly report here the definition of this property:

Definition 1. *A hash function h is Hamming-aware if there exist*

- *a set $\mathcal{Z}(k) \subseteq \Sigma^w$ such that $|\mathcal{Z}(k)| \in \mathcal{O}(c^k w^k)$, for some constant c, and*
- *a binary operation $\phi : \Sigma^w \times \Sigma^w \to \Sigma^w$ computable in $\mathcal{O}(w)$ time,*

such that if $P \in \Sigma^m$ then the following inclusion holds:

$$\{h(P') \ : \ P' \in \Sigma^m,\ d_H(P, P') \leq k\} \subseteq \{h(P)\ \phi\ z \ : \ z \in \mathcal{Z}(k)\} \tag{3}$$

Theorem 2. *The de Bruijn function $h_\oplus$ is a Hamming-aware hash function. In particular:*

- *The comparison binary operation for $h_\oplus$ is $\phi = \oplus$.*
- *$\mathcal{Z}(k) = \{h_\oplus(P_1) \oplus h_\oplus(P_2) : d_H(P_1, P_2) \leq k,\ P_1, P_2 \in \Sigma^m\}$ has $\mathcal{O}((2\sigma w)^k)$ elements.*

Proof. We prove the result for $\Sigma = \mathbb{Z}_2$, since each text on $\Sigma = \{0, ..., 2^r - 1\}, r > 0$ can be reduced to this case. We choose $\mathcal{Z}(k)$ to be the set

$$\mathcal{Z}(k) = \{h_\oplus(x) \oplus h_\oplus(y) \ : \ d_H(x, y) \leq k,\ x, y \in \Sigma^m\} \tag{4}$$

and the binary comparison operation to be $\phi = \oplus$. From this definition it is clear that, for every $x, y \in \Sigma^m,\ d_H(x, y) \leq k$ there will exist a $z \in \mathcal{Z}(k)$ such that $h_\oplus(x) = z \oplus h_\oplus(y)$ (more precisely, $z = h_\oplus(x) \oplus h_\oplus(y)$).

We use the same symbol $h_\oplus$ to indicate the matrix associated with the linear map $h_\oplus$. The following is an example that shows how such a matrix $h_\oplus$ looks like:

Example 1. Let $m = 16$, $w = 6$ and $\Sigma = \mathbb{Z}_2$. The matrix associated to the linear map ${}^m_w h_\oplus$ is

$$\begin{pmatrix}
\mathbf{1}&0&0&0&0&0&\mathbf{1}&0&0&0&\mathbf{1}&0&0&0&0&0\\
0&\mathbf{1}&0&0&0&0&0&\mathbf{1}&0&0&0&\mathbf{1}&0&0&0&0\\
0&0&\mathbf{1}&0&0&0&0&0&\mathbf{1}&0&0&0&\mathbf{1}&0&0&0\\
0&0&0&\mathbf{1}&0&0&0&0&0&\mathbf{1}&0&0&0&\mathbf{1}&0&0\\
0&0&0&0&\mathbf{1}&0&0&0&0&0&\mathbf{1}&0&0&0&\mathbf{1}&0\\
0&0&0&0&0&\mathbf{1}&0&0&0&0&0&\mathbf{1}&0&0&0&\mathbf{1}
\end{pmatrix}$$

In general, in the matrix associated with the linear map $h_\oplus$ each $h_\oplus[0, i],\ i = 0, w, 2w, ..., (\lceil m/w \rceil - 2) \cdot w, m - w$ is the upper element of a diagonal of ones.

The key observation is that there are a lot of identical columns in the matrix $h_\oplus$. For each $x, y \in \Sigma^m$ such that $d_H(x, y) \leq k$, $x \oplus y$ contains at most k ones so $z = h_\oplus(x) \oplus h_\oplus(y) = h_\oplus(x \oplus y)$ can be computed XOR-ing at most k columns of

the matrix $h_\oplus$. Intuitively, the elements of $\mathcal{Z}(k)$ can be computed XOR-ing at most k *distinct* columns of the matrix $h_\oplus$; since there are few distinct columns in $h_\oplus$, the size of $\mathcal{Z}(k)$ should then not blow-up with m, but with w instead. More formally, let

$$C_{h_\oplus} = \{x \in \Sigma^w \ : \ x \ is \ a \ column \ of \ h_\oplus\}$$

be the set of the columns of $h_\oplus$ (it is clear that this definition removes the duplicates). We want to prove the following:

Lemma 4.

$$\mathcal{Z}(k) = \{z \ : \ z = x_1 \oplus ... \oplus x_i, \ x_1, ..., x_i \in C_{h_\oplus}, \ i \le k\}$$

Proof.

$$\begin{aligned}\mathcal{Z}(k) &= \{h_\oplus(x) \oplus h_\oplus(y) \ : \ d_H(x,y) \le k, \ x, y \in \Sigma^m\} \\ &= \{h_\oplus(x) \oplus h_\oplus(y) \ : \ x \oplus y \ has \ at \ most \ k \ ones, \ x, y \in \Sigma^m\} \\ &= \{h_\oplus(x \oplus y) \ : \ x \oplus y \ has \ at \ most \ k \ ones, \ x, y \in \Sigma^m\} \\ &= \{h_\oplus(v) \ : \ v \ has \ at \ most \ k \ ones, \ v \in \Sigma^m\} \\ &= \{z \ : \ z \ is \ the \ XOR \ between \ at \ most \ k \ columns \ of \ h_\oplus\} \\ &= \{z \ : \ z = x_1 \oplus ... \oplus x_i, \ x_1, ..., x_i \in C_{h_\oplus}, \ i \le k\}\end{aligned}$$

□

Since $\mathcal{Z}(k)$ contains elements obtained XOR-ing at most k elements of $C_{h_\oplus}$, its size is

$$|\mathcal{Z}(k)| \in \mathcal{O}(|C_{h_\oplus}|^k)$$

We note that ${}^m_w h_\oplus$ is formed by $\lfloor m/w \rfloor - 1$ adjacent identity sub-matrices $I \in \Sigma^{w \times w}$ (one in the Example 1) and a final matrix of size $w \times (m - w(\lfloor m/w \rfloor - 1))$ having two diagonals of ones respectively starting in the upper left element and ending in the lower right element of the sub-matrix. Clearly, all the identity matrices contribute to exactly w distinct columns, while the last sub-matrix add at most other $w - 1$ distinct columns (formed only by zeroes except 2 ones separated by $m \ mod \ w - 1$ zeroes). It follows that $|C_{h_\oplus}| \le 2w$, so that we finally have

$$|\mathcal{Z}(k)| \in \mathcal{O}((2w)^k)$$

For a more general alphabet size $\sigma = 2^r, \ r > 0$ the above quantity becomes

$$|\mathcal{Z}(k)| \in \mathcal{O}\left((2(\sigma - 1)w)^k\right) = \mathcal{O}\left((2\sigma w)^k\right)$$

We note moreover that, if $x \oplus y$ has at most k ones, then $h_\oplus(x \oplus y)$ has at most $2k$ ones, so

Corollary 1. *If $\alpha, \beta \in \Sigma^m$ and $d_H(\alpha, \beta) \le k$, then $d_H(h_\oplus(\alpha), h_\oplus(\beta)) \le 2k$.*

□
