# Supplementary material for: Fast randomized approximate string matching with succinct hash data structures
Source: BMC Bioinformatics. 2015 Jun 1;16(Suppl 9):S4. doi: 10.1186/1471-2105-16-S9-S4 (PMC4464037; doi:10.1186/1471-2105-16-S9-S4)
Supplement: Additional file 2 — implementation usage file: additional file 2.pdf [file 1471-2105-16-S9-S4-S2.pdf]

Additional file 2 : implementation usage

Alberto Policriti[1,2] and Nicola Prezza[1]

[1] University of Udine, Department of Mathematics and Informatics, Udine, Italy
[2] Institute of applied genomics, Udine, Italy

1 Implementation usage: ERNE

We implemented our algorithm in the short reads aligner ERNE, to be used in DNA alignment (`http://erne.sourceforge.net`). Briefly, the implementation needs only two commands to perform alignment of a set of reads (in fastq format) against a reference genome:

1. **Index construction**: at the current version (1.4.2) ERNE implements both the classic hash index and the succinct dB-hash index described in this paper. The default structure used is the dB-hash; the user can specify –standard-hash to build the standard (old) hash data structure. To build the succinct version of our index call:

   ```
   erne-create --fasta ref.fasta --reference-prefix idx
   ```

   where `ref.fasta` is the input fasta file and `idx` is the prefix of the output index (extension will be automatically added to obtain `idx.ebh`). We recommend to allocate at least $3.5n$ Bytes of RAM (where n is the reference length) while building the index. Building the Human genome index requires approximately 6 hours and 9.5GB of RAM on a intel core i7, 2.4GHz machine. After construction, the index will require approximately $1.35n$ Bytes (and this will also be the RAM required for alignment).
2. **Alignment**: To align a fastq file to a indexed reference genome, just type

   ```
   erne-map --reference idx.ebh --query1 q.fq --output ali.bam
   ```

   erne-map automatically detects format of the index (standard hash/dB-hash) and produces output in standard bam format. For more details, please read the manual at `http://erne.sourceforge.net/manual.php`.
