# Supplementary material for: Fast randomized approximate string matching with succinct hash data structures
Source: BMC Bioinformatics. 2015 Jun 1;16(Suppl 9):S4. doi: 10.1186/1471-2105-16-S9-S4 (PMC4464037; doi:10.1186/1471-2105-16-S9-S4)
Supplement: Additional file 3 — commands used to perform the experiments file: additional file 3.pdf [file 1471-2105-16-S9-S4-S3.pdf]

Additional file 3 : commands used to perform the experiments

Alberto Policriti[1,2] and Nicola Prezza[1]

[1] University of Udine, Department of Mathematics and Informatics, Udine, Italy
[2] Institute of applied genomics, Udine, Italy

1 Creation of the indexes

bowtie

```
bowtie bowtie-build -f genome.fasta ref
```

BWA

```
BWA bwa index -a is genome.fasta
```

SOAP2

```
2bwt-builder genome.fasta
```

ERNE

```
erne-create --former-hash --fasta genome.fasta --k 15 --reference-prefix ref
```

BW-ERNE

```
erne-create --fasta genome.fasta --reference-prefix ref
```

2 Reads simulation

2.1 simseq

Sam simulation Note: *genome_haplotype2.fasta* is an alternative haplotype of *genome.fasta* used above for index creation

```
java -jar -Xmx4096m SimSeq.jar -1 100 \
                               -2 100 \
                               --error hiseq_mito_default_bwa_mapping_mq10_1.txt \
                               --reference genome_haplotype2.fasta \
                               --read_number 5000000 \
                               --out out.sam
```

Sam to fastq conversion

```
cat out.sam | grep -v ^@ | awk 'NR%2==1 {print "@"$1"\n"$10"\n+\n"$11}' >> query.fq
```

3 Alignments

ERNE

```
erne-map --sam \
         --reference ref.eht \
         --query1 query.fq \
         --output out.sam
```

Simseq and real datasets - BW-ERNE

```
erne-map --sam \
         --reference ref.ebh \
         --query1 query.fq \
         --output out.sam
```

GCAT data (no base qualities) - BW-ERNE

```
erne-map --sam \
         --sensitive \
         --no-auto-trim \
         --reference ref.ebh \
         --query1 query.fq \
         --output out.sam
```

Bowtie

```
bowtie bowtie -St ref query.fq out.sam
```

BWA

```
bwa aln genome.fasta query.fq > out.sai
bwa samse genome.fasta out.sai query.fq > out.sam
```

SOAP2

```
soap -a query.fq -D ref.index -o out
soap2sam.pl out >> out.sam
```

4 SNP simulation

Reads with simulated base qualities and SNPs were produced as follows:

1. SimSeq was used to generate reads with simulated base qualities
2. Using a simple script, each base was changed with probability 0.005 (1 SNP every 200 bp on average). For each selected base, the SNP type was chosen randomly (uniform distribution) among the 3 remaining bases.
3. Reads with SNPs were marked in the read name, to allow subsequent differentiated analysis.

5 Evaluation of the alignments

5.1 simseq

The evaluation followed these steps:

1. The first mate of each pair has been extracted from *out.sam* generated with simseq. Output has been stored in *out_1.sam*.
2. sam files (*out_1.sam* and those generated by the tools) have been sorted by read name
3. each sorted sam file has been compared with sorted *out_1.sam* counting the number of matches and taking into account read clipping in cigar vectors. An alignment was considered as correct if and only if all the following conditions were satisfied:
   - Same chromosome
   - Same strand
   - Mapping position within 50bp of simseq's position (to take into account indels and clips)
